# Experimental design data for the biosynthesis of citric acid using Central Composite Design method

**DOI:** 10.1016/j.dib.2017.03.049

**Published:** 2017-04-08

**Authors:** Anand Kishore Kola, Mallaiah Mekala, Venkat Reddy Goli

**Affiliations:** Department of Chemical Engineering, National Institute of Technology, Warangal, Telangana 506004, India

**Keywords:** Microbial production, Citric acid, Significant variables, Response surface methodology

## Abstract

In the present investigation, we report that statistical design and optimization of significant variables for the microbial production of citric acid from sucrose in presence of filamentous fungi *A. niger* NCIM 705. Various combinations of experiments were designed with Central Composite Design (CCD) of Response Surface Methodology (RSM) for the production of citric acid as a function of six variables. The variables are; initial sucrose concentration, initial pH of medium, fermentation temperature, incubation time, stirrer rotational speed, and oxygen flow rate. From experimental data, a statistical model for this process has been developed. The optimum conditions reported in the present article are initial concentration of sucrose of 163.6 g/L, initial pH of medium 5.26, stirrer rotational speed of 247.78 rpm, incubation time of 8.18 days, fermentation temperature of 30.06 °C and flow rate of oxygen of 1.35 lpm. Under optimum conditions the predicted maximum citric acid is 86.42 g/L. The experimental validation carried out under the optimal values and reported citric acid to be 82.0 g/L. The model is able to represent the experimental data and the agreement between the model and experimental data is good.

**Specifications Table**

**Table:** 

Subject area	*Biology*
More specific subject area	*Microbial biosynthesis*
Type of data	*Table, text file, graph, figure*
How data was acquired	*Analytical method*
Data format	*Analyze*
Experimental factors	*Determination of the yield of Citric acid*
Experimental features	*The sterilization for fermentation experiment was carried out for 25% sucrose solution by supplying the water and oxygen to the feremetror. After adding the nutrients, clear supernatant liquid was diluted to 15% sucrose level. The solution and growth medium were sterilized. The prepared culture was poured to fermentor and thereafter fermentation experiments were carried out for different operating conditions.*
Data source location	*Data of experimental and model provided in*Table 2.
Data accessibility	*Data provided in the article*

**Value of the data**

• The data presented for the production of citric acid using the *A. niger* using the batch solid state fermentor.
• The significant factors which influence on the growth of the citric acid are given in Table 3 both the CCD regression model and experimental data.
• The data presented in the article is useful to the industry as well as researchers. The detailed data of CCD and experimental data are useful to researchers for the production of citric acid with other strains.

## 1 Data

Citric acid is one of the most important biochemical products that are extensively used in many industrial processes like in the food technology to various fields of chemical industry [1]. Citric acid is in highly demandable product. It is produced by the extraction of citrus fruits, chemical synthesis and fermentation. Due to the limited supply of natural citric acid, it is produced commercially by using *A. niger* from the fermentation process of bulk hydrated materials and the byproducts of sugar production [2], [3], [4], [5].

Citric acid is produced mainly by *A. niger* from the fermentation process [6], [7]. The growth and production rate of *A. niger* are very much affected by the medium composition, fermentation variables and stimulators.

The worldwide demand for citric acid is increasing faster than its production and it requires more economical process models [3], [8]. The raw materials for citric acid production include; brewery wastes, corn starch, beet molasses, coconut oil, carob pod extract, glycerol date syrup, and pure sugars such as glucose and sucrose [9]. In addition to molds, several yeast strains are now known to produce large amounts of citric acid [10], [11].

The experimental plans were obtained from the Design expert soft ware. From that a set of combinations the experiments were performed. The experimental results were used to find a statistical mathematical model as a function of all the influenced factors or variables.

## 2 Experimental design, materials and methods

### 2.1 Materials and methods

*A. niger* NCIM 705 was purchased from National Chemical Laboratory, Pune, India. The substrate, sucrose, potato dextrose agar medium comprising dextrose, yeast extract and agar-agar for subculture preparation, growth medium consisting of glucose, NH_4_NO_3_, MgSO_4_·7H_2_O, KH_2_PO_4_, (NH_4_)SO_4_, Fe (SO_4_)_2_·24H_2_O and ZnSO_4_·7H_2_O, phenolphthalein indicator and 0.1 N NaOH for citric acid estimation [12] and di-nitro salicylic acid for sucrose estimation were procured M/s Hichem Chemicals, Warangal, India. Autoclave for sterilization of medium and fermentor, laminar flow chamber for inoculation and the glass fermentor of Scigenics India Ltd. make were used for experimentation.

### 2.2 Plackett–Burman design

The optimizations for the fermentation process are very important because of their influence on the economy and practicability of the process [13]. Plackett–Burman design is two level fractional factorial designs for N−1 variables in N experimental runs. The design considers only the main effects of the variables but not their interactive effects [14].

Citric acid yield is the response variable and the actual number of independent variables for the present system are eleven; initial sucrose concentration, methanol concentration, inoculums density, initial medium pH, spore age, stirrer rotational speed, incubation time, fermentation temperature, particle size distribution, oxygen flow rate and moisture content. Twelve experiments were recommended for eleven variables by Plackett–Burman design [15]. The twelve experiments were conducted accordingly to find the influence of each variable on the citric acid yield. If the variables have confidence levels greater than 95%, then their influence is more on the citric acid yield. From this design, it has been found that six most significant variables are namely initial sucrose concentration, initial medium pH, stirrer rotational speed, incubation time, fermentation temperature, and oxygen flow rate which strongly influence the citric acid yield*.*

### 2.3 Response Surface Methodology (RSM)

Response surface methodology is useful method for the modeling and analysis of all the industrial processes by using mathematical and statistical techniques. The output is influenced by various input variables. The main objective is to optimize the output by selecting the most significant variables which influence on the output or the response [15].

CCD method with most significant independent process variables (six variables) was used to find the effect of these variables on the yield of citric acid. From the CCD; sequential experiments are obtained for approximate information for testing the lack of fit [16], [17], [18], [19]. All the coded values are shown in Table 1. Hence the six factors investigated are initial sucrose concentration (X_1_), initial medium pH(X_2_), stirrer rotational speed(X_3_), incubation period(X_4_), fermentation temperature(X_5_), and oxygen flow rate(X_6_).

### 2.4 Experimental data

#### 2.4.1 Experimental setup and procedure

The pure culture of *A. niger* NCIM 705 was procured and preserved in a refrigerator by periodic subculture on potato dextrose agar medium. A fermentor capacity of 1.2 L contains a standard control and instrumentation panel. It was cleaned with pure water after that sterilized in an autoclave for 20 min. The sterilized fermentor was placed in the main assembly. The water as well as O_2_ is supplied by the tube connections. A 25% of sucrose solution was taken. After adding 35 mL of 1 N H_2_SO_4_, it was boiled for half an hour, cooled, neutralized with lime water and was left overnight for clarification. The clear supernatant liquid was diluted to 15% sucrose level. The solution and growth medium were sterilized, inoculated and the mixture was kept in an incubator for 24 h. The prepared culture was poured into the fermentor in the first run; thereafter the fermentor was put into operation for 7 days for batch operation [12].

#### 2.4.2 Analysis

All the samples were collected from the fermentor periodically for every 24 h and analyzed for sucrose, biomass and citric acid using standard analytical methods [20], [21].

#### 2.4.3 Statistical analysis

The Pareto analysis was used for the selection of a minimum number of tasks that gives a significant overall effect. The Pareto chart used for the contributory effect of each variable on citric acid fermentation.

After selecting the significant parameters; the CCD method used to find the effect of each significant parameter on the citric acid yield. A total of 86 sets of experiments were suggested by CCD with 79 being the combinations of the actual level of experimental variables while the remaining 7 were replications at the central points. The experiments were conducted as per the CCD method, to estimate the amount of citric acid produced. The experimental and predicted values of citric acid and the respective residual errors are shown in Table 2.

#### 2.4.4 Analysis of variance (ANOVA)

Table 3 shows the ANOVA results and regression analysis. The probability test values (*P*-values) less than 0.0500 indicates that the model terms are more significant on the citric acid yield. The higher the F value; better is the certainty, that the factors explain adequately the variation in the data about its mean and that the estimated factor effects are real. The ANOVA of the regression model demonstrates that the model is highly significant as is evident from a very high value of F (28.54) and a very low value of *P* (<0.0001). Based on p-value, it can be stated from the above Table 3 that initial sucrose concentration (X_1_), incubation time (X_4_), combined effect of initial sucrose concentration and stirrer rotational speed (X_1_ X_3_), combined effect of initial sucrose concentration and incubation time (X_1_ X_4_), square of the initial sucrose concentration $\left(X_{1}^{2}\right)$ and square of the stirrer rotational speed $\left(X_{3}^{2}\right)$ are significant model terms. Values greater than 0.1000 indicate that the model terms are not significant. *F*-value of 1.68 implies that the Lack of Fit is not significant relative to the error. There is a 20.55% chance that a large lack of fit *F*-value could occur due to noise. Non-significant lack of fit is desirable if the model is to be fit and it is true for the present study from the above Table 3. The predicted *R*^2^ is in reasonable agreement with the adjusted *R*^2^ (0.8974). It indicates that, the mathematical model is very reliable for the prediction of citric acid yield.

#### 2.4.5 The RSM curves

**Fig. 1:**
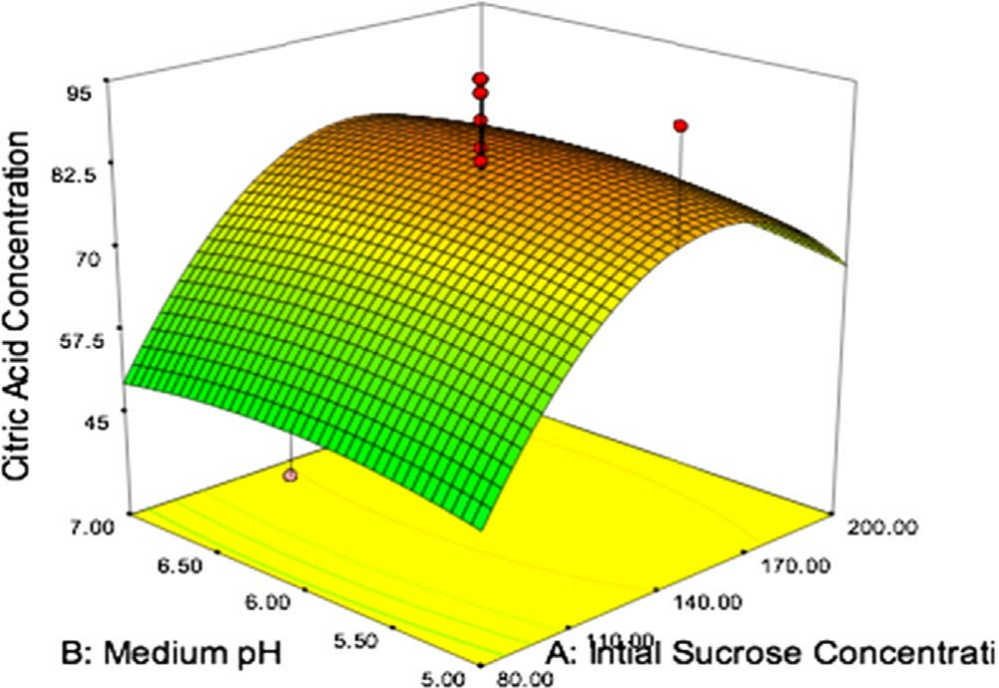
Yield of citric acid as a function of initial sucrose concentration and medium pH, while other four variables were kept constant at 240 rpm, 5.5 days, 30 °C and 1.5 lpm.

The citric acid yield response surface figure is shown in the Fig. 1. It gives the information that the yield of citric acid with initial concentration of the sugar and pH of the medium, the other variables are speed of 240 rpm, incubation time of 5.5 days, temperature of 30 °C and oxygen flow rate of 1.5 lpm. The maximum citric acid concentration is 86 g/L found at a pH of 6.26 and initial sucrose concentration of 140 g/L. The experimental citric acid yield obtained the present study is 82 g/L.

**Fig. 2:**
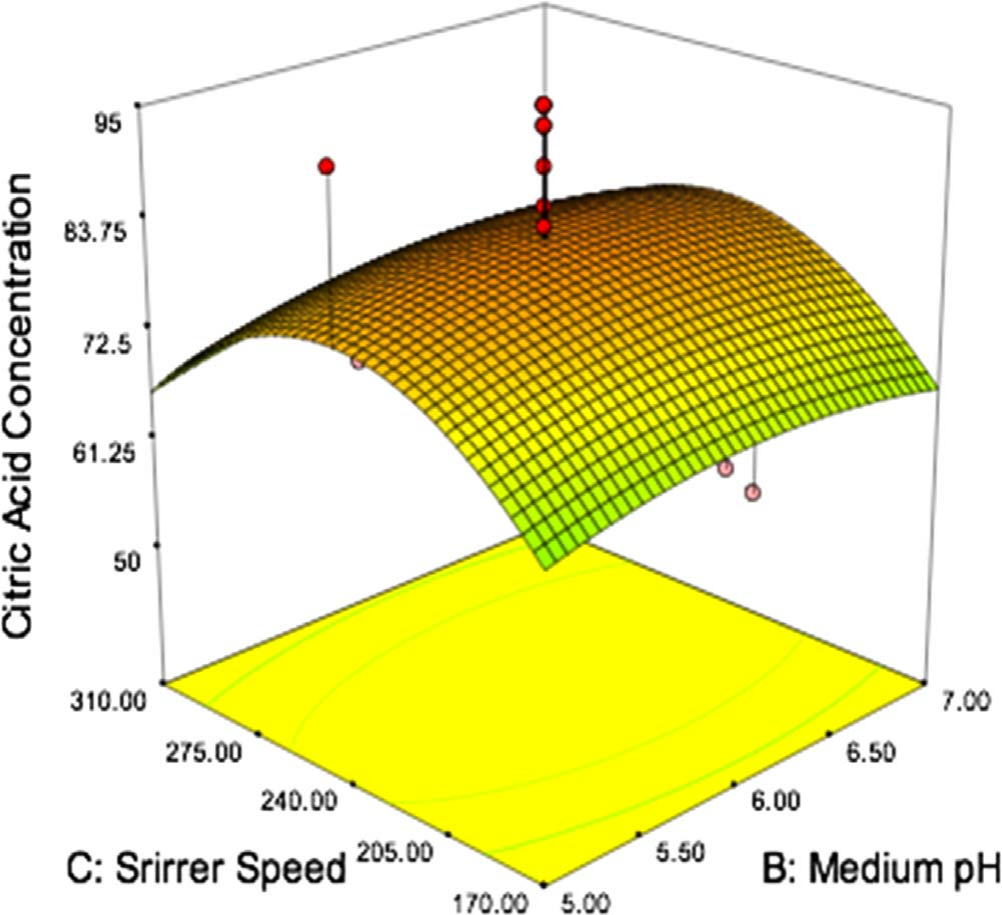
Yield of citric acid as a function of medium pH and stirrer speed, while the other four variables were kept constant at 140 g/L, 5.5 days, 30 °C and 1.5 lpm.

Fig. 2 shows the citric acid yield with respect to medium pH and stirrer speed and the other parameters are sucrose concentration of 140 g/L, incubation time of 5.5 days, temperature of 30 °C and oxygen flow rate of 1.5 lpm. The maximum predicted citric acid yield is 86 g/L.

## Figures and Tables

**Table 1:** Range of parameters selected for optimization.

Parameter	Code	Units	Low	High
Initial sucrose concentration	x_1_	g/L	80(−1)	200(+1)
Medium Ph	x_2_	–	5.0(−1)	7.0(+1)
Stirrer speed	x_3_	rpm	170(−1)	310(+1)
Incubation period	x_4_	days	1(−1)	10(+1)
Fermentation temperature	x_5_	°C	28(−1)	32(+1)
Oxygen flow rate	x_6_	lpm	0.5(−1)	2.5(+1)

**Table 2:** Design of experiments with experimental and predicted values of CA produced.

**Run no.**	**x**_**1**_	**x**_**2**_	**x**_**3**_	**x**_**4**_	**x**_**5**_	**x**_**6**_	**Experimental citric acid**	**Predicted citric acid**	**Residual value**
	**g/L**	**–**	**rpm**	**days**	**°C**	**lpm**	**g/L**	**g/L**	
1	80	5	170	1	28	0.5	16.5	25.364	−8.864
2	80	5	170	1	28	2.5	17.5	24.014	−6.514
3	200	5	170	10	28	2.5	46.1	50.184	−4.104
4	200	5	170	1	28	0.5	22	23.249	−1.249
5	80	7	170	10	28	0.5	15.3	17.875	−2.575
6	200	5	310	10	28	0.5	63.4	62.025	1.375
7	80	7	170	10	32	2.5	26.2	18.542	7.643
8	200	5	310	1	32	2.5	20	20.538	−0.538
9	80	5	310	10	28	2.5	10.4	10.865	−0.465
10	200	7	170	1	28	0.5	17.8	17.781	0.019
11	80	5	310	1	32	0.5	18.4	18.160	0.240
12	80	5	170	10	32	2.5	6.6	10.553	−3.953
13	80	7	170	10	28	2.5	20.8	15.108	5.692
14	200	5	170	1	32	2.5	19.5	17.420	2.054
15	200	7	310	1	28	2.5	21	22.701	−1.701
16	80	7	310	1	32	0.5	18.4	19.964	−1.564
17	80	5	310	1	28	0.5	19	18.063	0.937
18	200	5	170	10	32	0.5	46.3	47.639	−1.331
19	140	6	240	5.5	30	1.5	95	81.992	13.008
20	140	5	240	5.5	30	1.5	95	78.204	16.796
21	140	6	240	5.5	30	1.5	85	81.992	3.008
22	80	7	170	1	28	2.5	20	21.437	−1.437
23	80	5	170	1	32	2.5	17.5	20.995	−3.495
24	80	7	310	10	28	2.5	10.8	13.220	−2.420
25	80	7	310	1	28	2.5	18.2	13.530	4.670
26	80	7	310	10	32	2.5	10	19.947	−9.947
27	140	7	240	5.5	30	1.5	50	80.012	−30.012
28	200	5	310	10	28	2.5	52.5	56.028	−3.528
29	140	6	240	5.5	30	0.5	67.2	78.625	−11.455
30	200	6	240	5.5	30	1.5	65	71.665	−6.665
31	140	6	240	5.5	30	1.5	95	81.992	13.008
32	140	6	170	5.5	30	1.5	64	68.875	−4.875
33	200	7	310	1	32	2.5	22.4	26.333	−3.933
34	80	5	310	10	32	0.5	17.2	18.593	−1.393
35	140	6	240	5.5	30	1.5	93	81.992	11.008
36	80	5	310	1	28	2.5	18.2	15.013	3.165
37	200	5	310	10	32	0.5	62.8	58.476	4.324
38	200	7	310	1	28	0.5	22.8	20.401	2.399
39	200	7	170	1	32	0.5	23	17.942	5.058
40	200	7	170	1	32	2.5	24	22.120	1.880
41	80	5	170	1	32	0.5	24	22.167	1.833
42	80	7	310	1	28	0.5	19	13.140	5.860
43	200	7	170	1	28	2.5	22.7	21.781	0.919
44	200	5	310	1	28	0.5	22	24.774	−2.774
45	80	5	170	10	32	0.5	17.5	16.582	0.918
46	140	6	240	5.5	30	1.5	93	81.992	11.008
47	200	5	170	1	28	2.5	27.7	23.809	3.891
48	140	6	240	5.5	30	1.5	95	81.992	13.008
49	140	6	310	5.5	30	1.5	62	70.341	−8.341
50	200	7	170	10	32	2.5	45.6	52.058	−6.489
51	80	7	170	1	32	2.5	22.7	25.146	−2.446
52	200	7	310	10	32	2.5	88	62.289	25.711
53	140	6	240	10	30	1.5	70	79.770	−9.770
54	140	6	240	5.5	28	1.5	73	78.068	−5.068
55	80	7	310	10	32	0.5	28.4	24.236	4.164
56	200	5	170	10	28	0.5	58.6	54.481	4.119
57	80	5	310	10	28	0.5	20	18.772	1.228
58	200	7	170	10	28	2.5	51.1	51.994	−0.894
59	140	6	240	5.5	30	1.5	83	81.992	1.008
60	200	7	310	10	28	0.5	64	61.490	2.510
61	200	7	310	10	32	0.5	64.2	64.668	−0.468
62	80	5	310	10	32	2.5	10.4	10.864	−0.464
63	80	5	170	10	28	2.5	21.2	13.847	7.403
64	200	7	170	10	32	0.5	42	52.737	−10.737
65	80	7	310	1	32	2.5	19.8	20.533	−0.733
66	140	6	240	5.5	32	1.5	70	78.148	−8.148
67	200	7	310	1	32	0.5	22.4	23.855	−1.455
68	80	7	310	10	28	0.5	14.4	17.688	−3.288
69	140	6	240	5.5	30	2.5	75	76.761	−1.761
70	80	5	310	1	32	2.5	19.4	15.288	4.152
71	80	7	170	1	32	0.5	26.5	22.878	3.622
72	200	5	310	1	28	2.5	23	23.634	−0.634
73	200	7	310	10	28	2.5	55	58.933	−3.933
74	200	5	310	10	32	2.5	45	52.656	−7.656
75	80	6	240	5.5	30	1.5	45	51.551	−6.551
76	80	7	170	1	28	0.5	21.6	19.346	2.268
77	140	6	240	5.5	30	1.5	83	81.992	1.008
78	140	6	240	5.5	30	1.5	89	81.992	7.008
79	140	6	240	1	30	1.5	61	64.446	−3.446
80	200	5	170	1	32	0.5	20	16.682	3.318
81	140	6	240	5.5	30	1.5	75	81.992	−6.992
82	200	7	170	10	28	0.5	59	52.852	6.148
83	200	5	170	10	32	2.5	43.4	43.519	−0.136
84	80	7	170	10	32	0.5	26.6	21.131	5.469
85	200	5	310	1	32	0.5	16	21.500	−5.500
86	80	5	170	10	28	0.5	16.9	20.054	−3.154

**Table 3:** ANOVA and regression analysis.

Source	Sum of squares	Df	Mean square	*F*-value	*P*-value	Remarks
Model	56,935.14	27	2108.71	28.54	<0.0001	Significant
x_1_	6675.15	1	6675.15	90.35	<0.0001	–
x_2_	53.92	1	53.92	0.73	0.3964	–
x_3_	35.43	1	35.43	0.48	0.4914	–
x_4_	3874.26	1	3874.26	52.44	< 0.0001	–
x_5_	0.11	1	0.11	1.426E-003	0.9700	–
x_6_	57.37	1	57.37	0.78	0.3818	–
x_1_x_2_	1.21	1	1.21	0.016	0.8987	–
x_1_x_3_	311.60	1	311.60	4.22	0.0445	–
x_1_x_4_	5341.11	1	5341.11	72.30	< 0.0001	–
x_1_x_5_	45.44	1	45.44	0.62	0.4361	–
x_1_x_6_	14.59	1	14.59	0.20	0.6584	–
x_2_x_3_	4.79	1	4.79	0.065	0.7999	–
x_2_x_4_	58.93	1	58.93	0.80	0.3755	–
x_2_x_5_	181.05	1	181.05	2.45	0.1229	–
x_2_x_6_	47.34	1	47.34	0.64	0.4267	–
x_3_x_4_	144.89	1	144.89	1.96	0.1667	–
x_3_x_5_	11.56	1	11.56	0.16	0.6939	–
x_3_x_6_	11.56	1	11.56	0.16	0.6939	–
x_4_x_5_	0.30	1	0.30	4.108E−003	0.9491	–
x_4_x_6_	94.38	1	94.38	1.28	0.2630	–
x_5_x_6_	0.13	1	0.13	1.717E−003	0.9671	–
$x_{1}^{2}$	992.38	1	992.38	13.43	0.0005	–
$x_{2}^{2}$	19.87	1	19.87	0.27	0.6060	–
$x_{3}^{2}$	366.29	1	366.29	4.96	0.0299	–
$x_{4}^{2}$	233.33	1	233.33	3.16	0.0808	–
$X_{5}^{2}$	36.03	1	36.03	0.49	0.4877	–
$X_{6}^{2}$	44.14	1	44.14	0.60	0.4427	–
Residual	4284.92	58	73.88	–	–	–
Lack of Fit	3862.52	49	78.83	1.68	0.2055	not significant
Pure Error	422.40	9	46.93	–	–	–
Corr Total	61,220.05	85		–	–	–
