# Supplementary material for: Experimental design data for the biosynthesis of citric acid using Central Composite Design method
Source: Data Brief. 2017 Apr 8;12:234–41. doi: 10.1016/j.dib.2017.03.049 (PMC5397574; doi:10.1016/j.dib.2017.03.049)
Supplement: Supplementary file 1 — Supplementary material [file mmc1.docx]

**Conflicts of Interest**

The authors have to declare that no conflicts of interest.
